# Impact of nitrogen (N) and phosphorus (P) enrichment and skewed N:P stoichiometry on the skeletal formation and microstructure of symbiotic reef corals

**DOI:** 10.1007/s00338-022-02223-0

**Published:** 2022-04-20

**Authors:** M. C. Buckingham, C. D’Angelo, T. B. Chalk, G. L. Foster, K. G. Johnson, Z. Connelly, C. Olla, M. Saeed, J. Wiedenmann

**Affiliations:** 1 School of Ocean and Earth Science (SOES), University of Southampton, Southampton, UK; 2 Natural History Museum, London, UK

**Keywords:** *Acropora*, Nutrient enrichment, Stoichiometry, Linear extension, Calcification, Skeletal density

## Abstract

Reported divergent responses of coral growth and skeletal microstructure to the nutrient environment complicate knowledge-based management of water quality in coral reefs. By re-evaluating published results considering the taxonomy of the studied corals and the N:P stoichiometry of their nutrient environment, we could resolve some of the major apparent contradictions. Our analysis suggests that Acroporids behave differently to several other common genera and show distinct responses to specific nutrient treatments. We hypothesised that both the concentrations of dissolved inorganic N and P in the water and their stoichiometry shape skeletal growth and microstructure. We tested this hypothesis by exposing *Acropora polystoma* fragments to four nutrient treatments for > 10 weeks: high nitrate/high phosphate (HNHP), high nitrate/low phosphate (HNLP), low nitrate/high phosphate (LNHP) and low nitrate/low phosphate (LNLP). HNHP corals retained high zooxanthellae densities and their linear extension and calcification rates were up to ten times higher than in the other treatments. HNLP and LNLP corals bleached through loss of symbionts. The photochemical efficiency (Fv/Fm) of residual symbionts in HNLP corals was significantly reduced, indicating P-starvation. Micro-computed tomography (µCT) of the skeletal microstructure revealed that reduced linear extension in nutrient limited or nutrient starved conditions (HNLP, LNHP, LNLP) was associated with significant thickening of skeletal elements and reduced porosity. These changes can be explained by the strongly reduced linear extension rate in combination with a smaller reduction in the calcification rate. Studies using increased skeletal density as a proxy for past thermal bleaching events should consider that such an increase in density may also be associated with temperature-independent response to the nutrient environment. Furthermore, the taxonomy of corals and seawater N:P stoichiometry should be considered when analysing and managing the impacts of nutrient pollution.

**Supplementary Information:**

The online version contains supplementary material available at 10.1007/s00338-022-02223-0.

## Introduction

Nutrient enrichment through the introduction of excess nitrogen and/or phosphorus in reef environments typically promotes an array of direct and indirect negative effects leading to the decline of zooxanthellate coral cover. Impacts include increased susceptibility of corals to bleaching, disease and bio-erosion, greater competition for light and space from algal competitors and the increased abundance of corallivores (Kinsey and Davies 1979; Edinger et al. 2000; Brodie et al. 2005; Fabricius 2005; D’Angelo and Wiedenmann 2014; Vega Thurber et al. 2014). Nutrient enrichment can also impact coral skeletal growth and structure. However, different studies report contradictory results regarding how changes in the nutrient environment affect linear extension, calcification and skeletal structure (Koop et al. 2001; Fabricius 2005; Dunn et al. 2012; Shantz and Burkepile 2014; Szmant 2002; Tomascik and Sander 1985; Rocker et al. 2017; Marubini and Davies 1996). This uncertainty is of concern as the nutrient environments in coral reefs are likely to undergo continued change due to direct anthropogenic impact and climate change. Such changes may occur in the form of nutrient enrichment (Elizalde-Rendón et al. 2010; Browne et al. 2015), skewed N:P stoichiometries (D'Angelo and Wiedenmann, 2014; Lapointe et al. 2019) or nutrient depletion (Sun et al. 2008; Rosset et al. 2017). Accordingly, varied effects on coral skeletons can be expected, which may influence the formation of the 3-dimensional reef framework that is critically important for reef biodiversity and productivity (Purkis et al. 2008; Graham and Nash 2013), and coastal protection (Sheppard et al. 2005). Furthermore, impaired coral growth and changes in skeletal structures may shift the reef accretion/erosion balance towards net erosion (Lange and Perry 2019; Perry et al. 2020) and the consequent loss of rugosity may negatively affect ecosystem services such as fisheries, tourism income and coastal protection. Paradoxically, even when nutrient enrichment may promote coral growth, this can occur alongside reliable indicators of reef degradation such as reduced live coral cover and increased rates of bio-erosion (Edinger et al. 2000). This incomplete understanding impairs knowledge-based management of the nutrient environment in coral reefs and may prevent stakeholder support for required coastal zone and catchment management projects (Bell et al. 2007). Therefore, a better understanding of the impacts of changes in the nutrient environment on skeletal growth and structure is needed to forecast, and potentially mitigate, effects of environmental change on reef ecosystems.

Coral skeletal growth is commonly quantified using three metrics: linear extension, calcification rates and skeletal density. Linear extension describes the change in length of branches or foliose skeletons, or the increase in diameter of massive species. Calcification refers to the precipitation of the aragonite (CaCO_3_) skeleton. In many experiments, calcification is commonly presumed to be reflected mostly in a mass change as the contribution of the soft tissue to the overall weight is relatively small. Skeletal density is often measured alongside growth and is ultimately a property of the skeletal microstructure. Skeletal density comprises two components: micro-density and bulk density. Micro-density refers to the specific gravity of the material from which the skeleton is formed and is affected by the inclusion of trace elements, non-CaCO_3_ compounds and organic content; bulk density is the mass divided by the total volume and takes into account the micro-density and the porosity of the skeletal structure (Bucher et al. 1998; Caroselli et al. 2011). Porosity is the primary control on bulk density (to which it is negatively correlated) and is important ecologically because high skeletal porosity is associated with reduced mechanical strength and greater susceptibility to breakage and erosion caused by biotic and abiotic factors (Chamberlain, Jr. 1978; Bucher et al. 1998; Marshall 2000).

Coral growth is limited by the availability of energy and nutrients, mostly in the form of carbon, nitrogen and phosphorus (Dubinsky and Jokiel 1994; Davy et al. 2012; D’Angelo and Wiedenmann 2014; Rädecker et al. 2015; Ferrier‐Pagès et al*.*, 2016). At a global scale, mean concentrations of dissolved nitrate (0.25 $\pm 0.28 \mu M)$ and phosphate (0.13 $\pm 0.08 \mu M)$ in coral reef waters are relatively low (Kleypas et al. 1999). Nitrogen is most commonly the limiting nutrient on coral reefs (Kleypas et al. 1999; Furnas et al. 2005, D'Angelo and Wiedenmann, 2014). These low nutrient concentrations limit water column productivity and, subsequently, the availability of coral food. Consequently, reef-forming corals rely on a mutually beneficial symbiosis with microscopic dinoflagellates of the family *Symbiodiniaceae* (LaJeunesse et al. 2018) (commonly referred to as zooxanthellae) to access nutrients in their dissolved inorganic forms that are otherwise not accessible to the coral animals (Falkowski et al. 1984, 1993; Davy et al. 2012, Rädecker et al. 2015; Ferrier‐Pagès et al*.*, 2016). The photosynthetic symbionts—which reside in the coral gastrodermis—translocate excess carbohydrates to the coral, in some cases providing > 90% of the host’s energetic requirements (Falkowski et al. 1984, 1993). ^15^N tracer studies have demonstrated the capacity of the symbionts to incorporate NO_3_^−^ and subsequently translocate the labelled N (Grover et al. 2003; Tanaka et al. 2006). Aposymbiotic and non-symbiotic corals are incapable of incorporating dissolved PO_4_^3−^, while in species harbouring zooxanthellae, PO_4_^3−^ uptake increases in the light presumably in association with photosynthesis (reviewed in Davy et al. 2012; Ferrier‐Pagès et al*.*, 2016).

The importance of the symbiosis for coral growth is demonstrated by the fact that calcification rates may be up to 4 times higher in the light compared to the dark and these increases coincide with elevated symbiont photosynthesis and host respiration (Furla et al. 2000). However, enhanced zooxanthellae density and photosynthesis associated with nutrient enrichment may occur alongside increases (Koop et al. 2001; Dunn et al. 2012) or decreases (Fabricius 2005; Shantz and Burkepile 2014) in skeletal growth. Nutrient enrichment at skewed N:P ratios can have a detrimental effect on symbiotic corals as the relative oversupply of one nutrient leads to nutrient starvation of zooxanthellae with respect to another (Wiedenmann et al. 2013; Rosset et al. 2017). In particular, N-enrichment without sufficient supply with phosphorus stimulates the unsustainable proliferation of zooxanthellae facilitated by the reallocation of cellular P resources by the symbionts to support vital metabolic processes. This ultimately causes P-starvation of the zooxanthellae, reduces the host’s resistance to heat and light induced bleaching and decreases coral biomass (Wiedenmann et al. 2013; Rosset et al 2017). Contrastingly, the experimental addition of P, along with N, can ameliorate the negative effects of N enrichment alone (Shantz and Burkepile 2014).

The impact of skewed N:P ratios on polyp size and biomass (Rosset et al. 2017) suggests that skeletal growth may be similarly impacted. We therefore categorised published studies on skeletal growth and structure with a consideration of the N:P stoichiometry experienced by the corals. With this approach, we could resolve some of the major apparent contradictions of previous studies. Notably, the taxonomy of the corals under study seems to be an important determinant in shaping the effect of the nutrient environment on skeletal parameters, with members of the genus *Acropora* responding often differently compared to representatives of several other genera. To test the resulting hypothesis that skewed N:P ratios can alter skeletal growth and micro-structure, we cultured replicate colonies of *Acropora polystoma* associated with *Cladocopium* sp. symbionts under a suite of different nutrient regimes comparing the effects of skewed N:P stoichiometries, nutrient replete and nutrient-limited conditions. The effects on coral growth and symbiont physiology were assessed along with differences in the skeletal microstructure determined by micro-computed tomography ($\mu$-CT).

## Methods and materials

### Analysis of published studies

We collated 92 coral responses from 25 papers (Supplementary Table 1 and Supplementary References) which reported the impacts of seawater nutrient concentrations on the most commonly used skeletal growth metrics: linear extension, calcification and skeletal density. We categorised nutrient enrichment scenarios into three regimes, solely according to the relative molar concentrations of N and P: high nitrogen: low phosphorus (HNLP) where N:P > 35 and low nitrogen:high phosphorus (LNHP) where N:P < 0.5. The corals were considered to be exposed to HNHP or nutrient replete conditions when concentrations of both N and P were higher than the global average ( ^~^0.25 $\pm 0.28 \mu$ M NO_3_^−^ $,$ ~0.13 $\pm 0.08$ $\mu$M PO_4_^3−^) (Kleypas et al. 1999) and available at N:P ratios between 0.5 and 35. Importantly, our categorisation of nutrient environments included all species of DIN reported by the original authors. Consequently, where concentrations of NH_3_/NH_4_^+^ were available our quantification of N:P ratios typically exceed the “global average” of Kleypas et al. (1999) who-only considered NO_3_^−^ and PO_4_^3−^. To ensure the most reliable assessment of the literature, all non-significant effects reported by the original authors were categorised alongside reports of no effect as ‘No effect’. Thus, any ‘Increase’ or ‘Decrease’ reported in our review refers only to statistically significant effects as reported by the authors of the original publication.

### Coral husbandry

Coral colonies were cultured in the experimental mesocosm at the National Oceanography Centre, Southampton, UK, which is described in detail in D’Angelo and Wiedenmann (2012). Temperature (~27 °C) and salinity (~33psu) were maintained at constant levels and a 12-h light/dark cycle at a surface light intensity of ~125 mol$\mu$ m^−2^ s^−1^ was provided by metal halide lamps (Aqualine 10,000, Aqua Medic, Germany). Each experiment used genetically identical replicate colonies (~20–25 mm) from a single parent colony of *A. polystoma* attached to ceramic tiles using epoxy resin. Following fragmentation, corals were allowed to recover for > 3 weeks before being exposed to four dissolved inorganic nutrient treatments that were previously used to simulate nutrient replete and strongly nutrient limited conditions as well as skewed N:P stoichiometry (Rosset et al. 2017). Specifically, the long-term nutrient regimes over the duration of the experiment in the different experimental system were as follows: high nitrate/high phosphate (HNHP, NO_3_^−^ ~4.5 $\mu \mathrm{M},$ PO_4_^3−^ ~0.6$\mu \mathrm{M}$, N:P ~8:1), high nitrate/low phosphate (HNLP, NO_3_^−^ ~0.073 $\mathrm{mM},$ PO_4_^3−^ not detectable (method detection limit = 0.21 $\mu \mathrm{M}$), low nitrate/high phosphate (LNHP, NO_3_^−^ ~0.06 $\mu \mathrm{M},$ PO_4_^3−^ ~5.7 $\mu \mathrm{M}$, N:P ~0.01) and low nitrate/low phosphate (LNLP, NO_3_^−^ not detectable$,$ PO_4_^3−^$\text{not\ detectable})$.

The terms ‘high’ and ‘low’ describe the relative concentrations of NO_3_^−^ and PO_4_^3−^ in our treatments as detailed previously (Wiedenmann et al. 2013; Rosset et al. 2017). The N and P concentrations of our HNHP treatment are in the range of those found on high nutrient reefs environments at the Galápagos Islands and off the Brazilian coast or in reefs subject to internal wave-driven upwelling (Aston et al. 2019; Kleypas et al. 1999; Szmant 2002). Meanwhile, the respective N and P concentrations of the HNLP and LNHP treatments exceed levels observed on unpolluted reefs. The experiment was repeated three times. Corals were not fed during the experiments. Nutrient concentrations were adjusted by the addition of NaNO_3_ and NaPO_4_^3−^ solutions if required, ammonium levels in these systems are constantly low (Wiedenmann et al. 2013). Nitrate in the LNLP conditions was removed continuously from the systems by use of Nitrate reactors (Aqua Medic, Germany). Phosphate in the HNLP and LNLP treatments was removed by filtering the water through RowaPhos Matrix (D-D The Aquarium Solution Ltd, UK). Nutrient concentrations were monitored weekly using the colourimetric detection methods with HACH DR900 Colourimeter (Hach, USA) described in detail in Rosset et al. (2017). The positions of corals were regularly alternated in the tanks to minimise any random effects due to light and/or water flow.

### Measuring skeletal growth, photosynthetic efficiency, zooxanthellae density and bleaching

Linear extension of the corals along the main axis and side branches was measured using calipers (accuracy $\pm 0.1 \mathrm{mm})$. The position of the main axial corallite at the start of the experiment was subsequently used to determine the boundary between ‘old’ and ‘new’ skeleton. Mass change was determined from wet weight after a defined drip-off period and removal of any non-coral growth from the attachment tile as described in Rosset et al. (2017). Since mass gain in Acroporids is dominated by the deposition of skeletal material, the terms calcification and mass gain are used interchangeably hereafter. The maximum quantum efficiency of PSII photochemistry (Fv/Fm) of zooxanthellae was measured using a submersible pulse amplitude modulated fluorometer (Diving-PAM, Walz, Germany) after > 10 h dark acclimation at minimal background light levels. Zooxanthellae density was determined using a haemocytometer following the removal of the host tissue with a waterpick and subsequent separation of host and symbiont fraction by differential centrifugation (Rosset et al. 2017). The visual bleaching response of corals over time was recorded by a single observer using a CoralWatch$\copyright$ colour card, with a decrease in colour score $\ge$ 2 being considered a bleaching response (Siebeck et al. 2006).

### Analysis of skeletal growth using calcein staining

Prior to the 73-d culture, corals were incubated under HNHP conditions in seawater containing calcein (Sigma-Aldrich, Germany) solution at a concentration of ~100 $\mu$ M for 72 h according to the staining protocols detailed in Tambutté et al. (2011) and Ohno et al. (2017). Corals were then soaked twice for 30 min in clean seawater to rinse calcein from the tissue surface and prevent contamination of the experimental mesocosm before being placed into their respective treatment compartments. On completion of the experimental exposure, fragments were first frozen before the tissue was removed using a Waterpick. Subsequently, the skeletons were washed twice in 10% NaClO (Sigma-Aldrich, Germany) solution for 30 min to remove any residual organic matter before being thoroughly rinsed in MilliQ water (18.2 M ohm cm) and then oven dried. Fragments were then embedded in epoxy resin, cut into 50 $\mathrm{\mu M}$ thick cross-sectional slabs using a slow speed saw and polished using silicon carbide paper. Calcein staining patterns were documented by photographing the thin section under a MZ10 Fluorescent Stereo Microscope (LEICA Microsystems, UK), using a Green Fluorescent Protein longpass filter. The fluorescence micrographs were stitched together to cover the full region of interest (ROI). Staining patterns were emphasised by enhancing the red image channel (showing unstained skeleton) and green image channel (showing stained skeleton) using Adobe Photoshop. The blue image channel was set to black.

### Micro-CT scanning

Skeletons were cleaned and dried as described above. Scanning was conducted at The University Hospital Southampton, UK (UHS), using the Nikon Med-X (alpha) prototype (Nikon X-Tek Systems Ltd, UK) and at The Natural History Museum, London, UK (NHM), using a Nikon Metrology HMX ST225 (Nikon Metrology, Tring, UK). Fragments were analysed at UHS using a beam with voltage of 95 kV and 116 $\mu$A current was generated using a Tungsten reflection target and a 0.25 mm aluminium filter. At the NHM fragments were scanned using a beam with voltage of 100 kV. A 100 $\mu$A current was generated with a Tungsten reflection target and a 0.5 mm aluminium filter. In both cases, projections were obtained for each sample during a single 360° rotation and each set of radial projections was subsequently reconstructed into a 3-dimensional matrix of isotropic voxels (at a resolution of 11.5 μm for UHS samples and 12.5 μm for NHM samples) using CT Pro 3D v5.

### Thickness and porosity of skeletal elements

When analysing differences in skeletal microstructure, we distinguished between ‘old’ skeleton grown prior to the start of the experimental treatment and ‘new’ skeleton that grew under controlled treatment conditions. The position of the axial polyp in relation to the base was recorded at the start of each experiment. ‘Old skeleton’ was defined as the skeleton present below this point at the conclusion of the growth experiment; ‘new skeleton’ was defined as the skeleton which had formed during the experiment above the original tip of the axial corallite. The skeletal microstructure of *A. polystoma* comprises an axial corallite from which numerous radial corallites diverge. The coenosteum (the skeleton between corallites) is formed from a lattice of interconnected synapticular ‘bars” and trabecular “rods” (Gladfelter 2007; Humblet et al. 2015). The axial and radial corallites comprise a central cavity that contains diminutive septae. When characterising the thickness of the skeletal element, we have not distinguished between these different components in our analysis and from hereon we refer to all skeletal components collectively as ‘skeletal elements’.

$\mu$-CT images were analysed using ImageJ (Fiji) version 2.0.0. For each fragment, regions of interest (ROI’s) were selected for analysis. ROI’s comprised a 0.25 mm thick planar cross section perpendicular to the direction of axial growth. ‘Old skeleton’ and ‘new skeleton’ ROI’s were located ~0.75 to ~0.50 mm below, and ~0.50 to ~0.75 mm above the original axial corallite tip, respectively (Fig. 4a). Measurements of mean skeletal thickness, skeletal volume (SV) and total skeletal volume (TV) were obtained using the BoneJ plugin. Porosity was subsequently calculated as: Porosity (%) = 1- $\frac{SV}{TV}$ × 100. All slices within selected ROI’s were measured individually to ensure that an identified artefact of bulk measurement did not influence the absolute measured values. Changes in mean skeletal element thickness and porosity with distance from the corallite tip were determined from measurements of 1-voxel thick slices at 0.25 mm intervals in the upper 9 mm of the fragments from the 100-d culture only. A macro was used to ensure faithful replication of the method for each set of measurements which is available in the supplementary material online.

### Statistical analysis

Statistical analysis was conducted using R (version 4.0.3). One-way ANOVA and Tukey Honestly Significant Difference tests were used to determine differences between treatments. Where the underlying assumptions regarding equality of variability and normality of distribution were not met, Kruskal–Wallis test was favoured and Dunn’s test was employed to determine differences between treatments. Corals analysed using $\mu$-CT images were selected from the 100 and 140 d cultures, respectively. No significant differences were detected between the two datasets (stepwise Welch’s t-tests) so data were pooled. The relationship between skeletal element thickness and porosity to distance from the corallite tip were assessed using Pearson’s correlation coefficient.

## Results

### Analysis of published studies

**Fig. 1:**
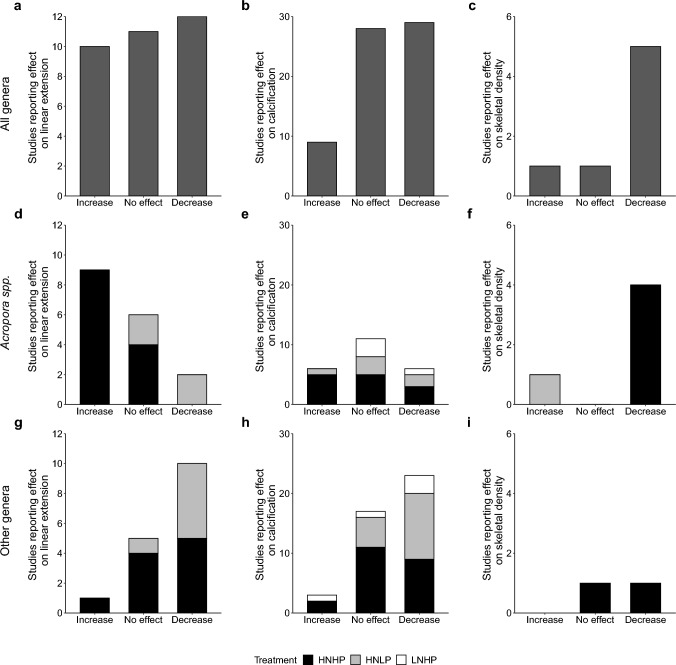
Analysis of 25 published research papers covering 92 comparisons (studies) of changes in linear extension, calcification and skeletal density to changes in the dissolved inorganic nutrient environment. **a**–**c** Reported effects from all studies with no distinction between the genus of the studied coral species or the stoichiometry of the nutrient environment. **d**–**f** Reported effects from 33 studies from 8 publications involving 10 species of Acroporids. **g**–**i** Reported effects of 6 genera of non-*Acropora* genera from 59 studies in 23 papers. “Other genera” include *Porites* spp., *Stylophora* spp., *Pocillopora* spp., *Montastrea* spp., *Montipora* spp. and *Merulina* spp. HNHP = high nitrate: high phosphate (N:P = 0.5–35), HNLP = high nitrate: low phosphate (N:P > 35), LNHP = low nitrate: high phosphate (N:P < 0.5). All increases and decreases to skeletal parameters refer to statistically significant (*p* < 0.05) findings, whereas “no effect” results include increases and decreases deemed non-significant (p > 0.05)

The reviewed studies encompassed seven different coral genera and a range of nutrient enrichment scenarios including both field and laboratory settings (see supplementary material). When the responses are grouped disregarding the taxonomy of the studied corals and the type of nutrient enrichment, the only clear trend is a decrease in skeletal density (Fig. 1a–c). Regarding linear extension and calcification rates, more studies showed no effects or increases than decreased rates. However, when the results of these studies were categorised according to the N:P stoichiometry and coral taxonomy (*Acropora* v other genera), responses of *Acropora* spp. were clearly different (Fig. 1d–f). Across a range of different settings, nutrient enrichment caused linear extension in *Acropora* spp. to increase under HNHP conditions but resulted in a decrease or ‘no effect’ under HNLP conditions. In *Acropora* spp., the impact on calcification was more variable but increases were commonly reported under HNHP conditions. Skewed nutrient ratios were generally associated with ‘no effect’ or decreases. Under HNHP conditions, the skeletal density of *Acropora* spp. decreased. In contrast, for other genera, linear extension and calcification commonly decreased following nutrient enrichment regardless of N:P stoichiometry, but there was no consistent impact on skeletal density (Fig. 1g–i). The high number of studies reporting increased linear extension and/or calcification in *Acropora* spp. under HNHP conditions suggests that when the availability of both N and P is elevated, skeletal growth in this genus is enhanced. In contrast, whenever N:P ratios exceed ~72:1, the linear extension and calcification of *Acropora* spp. are more likely to be reduced, suggesting that the relative undersupply of P inhibits skeletal growth.

### Nutrient effects on *Acropora polystoma* determined in controlled laboratory experiments

#### Effects of nutrient treatments on zooxanthellae density and photosynthetic efficiency

**Fig. 2:**
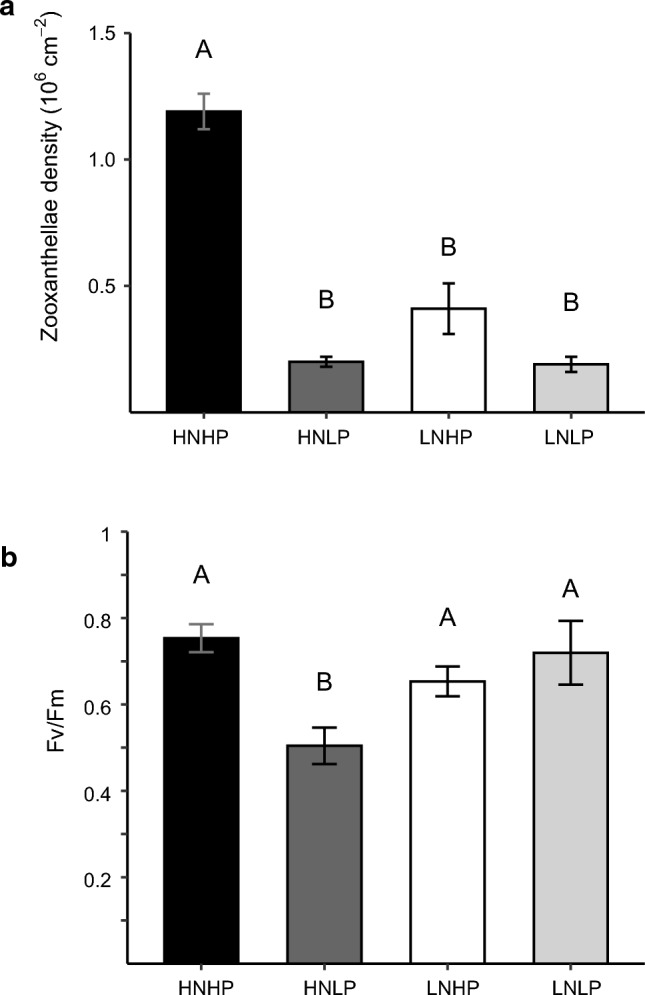
Symbiont densities and their photosynthetic efficiency in *A. polystoma* after exposure to different nutrient treatments for 140 d *.* **a** Mean zooxanthellae densities of *A. polystoma* fragments (ANOVA, *p* = 0.0003, *n* = 2). **b** Quantum efficiency of Photosystem II (Fv/Fm) of *A. polystoma* (ANOVA, *p* = 0.001, *n* = 4). HNHP = high nitrate: high phosphate, HNLP = high nitrate: low phosphate, LNHP = low nitrate: high phosphate and LNLP = low nitrate: low phosphate. Letters above bars indicate significant differences between treatments

All replicate colonies of *A. polystoma* cultured in the HNHP treatments remained unbleached, while all corals cultured in the HNLP and LNLP treatments bleached. At the conclusion of the 140-d culture experiment, the zooxanthellae density in HNHP corals was ~3 times higher (~1.2 × 10^6^ cm^−2^) than in those from the LNHP treatment (~0.4 × 10^6^ cm^−2^) and ~sixfold higher than in corals from the HNLP and LNLP treatments (~0.2 × 10^6^ cm^−2^) (ANOVA, F_3_ = 100.8, *p* = 0.0003) (Fig. 2a). Fv/Fm was significantly reduced in corals from the HNLP treatment when cultured for 140 d (ANOVA, F_3,8_ = 15.4, *p* = 0.001) (Fig. 2b), but corals retained high values of Fv/Fm in the other nutrient treatments.

#### Effects of nutrient treatments on skeletal growth

**Fig. 3:**
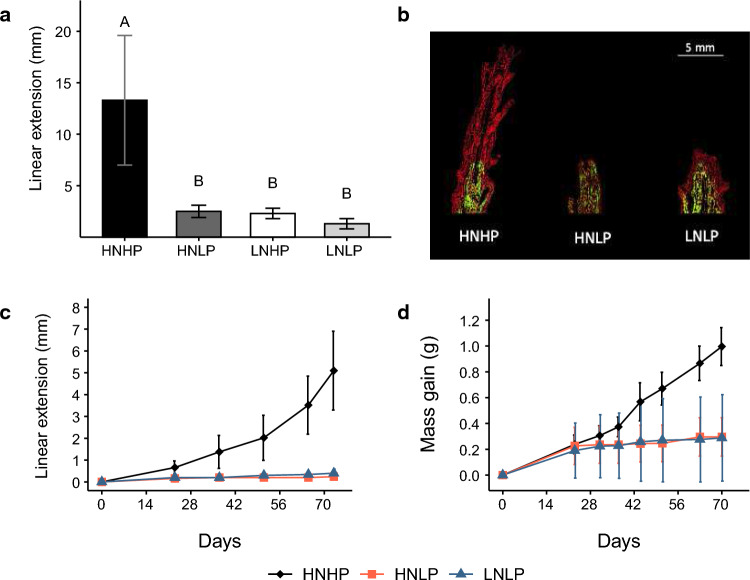
Skeletal growth of *A. polystoma* in different nutrient environments. **a** Linear extension of *A. polystoma* after 140-d culture (ANOVA, *p* = 0.0006, *n* = 4). Letters indicate significant differences between treatments. **b** Fluorescence micrographs of longitudinal cross sections of calcein-stained *A. polystoma* skeletons imaged with a digital camera fitted to a Leica Mz10 Fluorescent Stereo Microscope using a GFP longpass filter. Calcein stained skeleton highlighted by the green fluorescence. Newly deposited skeleton appears in red. Time course measurements of **c** Linear extension (mean $\pm$ s.dev.) and d) mass gain (mean $\pm$ s.dev.) of *A. polystoma* over 73-d culture in different nutrient environments (*n* = 5). HNHP = high nitrate: high phosphate, HNLP = high nitrate: low phosphate, LNHP = low nitrate: high phosphate and LNLP = low nitrate: low phosphate

After 140 d, corals cultured in the HNHP treatment extended ~5-times more than those under the other treatments (ANOVA, F_3,12_ = 12.18, *p* = 0.0006) (Fig. 3a). Analysis of the calcein stained skeletons confirmed the differences in the skeletal growth between treatments; the extensive formation of ‘new’ (unstained skeleton) at the tips of the HNHP corallites that was largely absent from corals cultured in the HNLP and LNLP treatments (Fig. 3b). The relationship between linear extension and mass gain was further investigated in a 73-d culture experiment. Linear extension and mass gain of HNHP corals were ~ tenfold and ~ threefold higher, respectively, compared to their counterparts from HNLP and LNLP treatments (Kruskal–Wallis: linear extension *p* = 0.006, mass gain *p* = 0.01). Notably, the greater extent to which linear extension was affected relative to mass gain in the HNHP corals can be explained by the fact that linear extension increased at an exponential rate whereas the mass showed an approximately linear increase (Fig. 3c and d).

#### Effects of nutrient treatments on skeletal microstructure

**Fig. 4:**
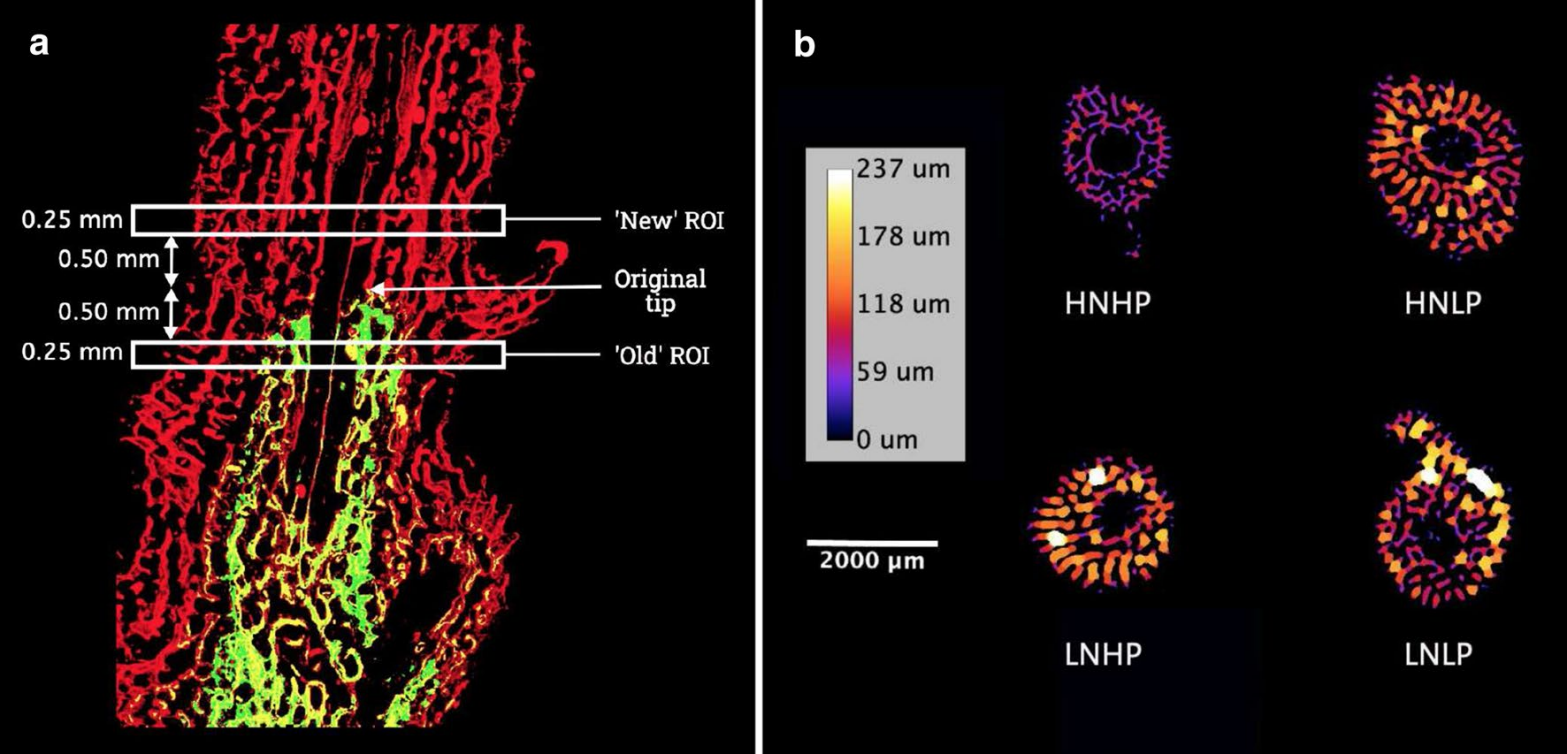
Skeletal microstructure of *A. polystoma* from different nutrient environments. **a** Fluorescence micrograph of longitudinal cross sections of calcein stained *A. polystoma* skeletons imaged with a digital camera fitted to a Leica Mz10 Fluorescent Stereo Microscope using a GFP longpass filter. Calcein stained skeleton appears green and newly deposited skeleton appears red. The selection of regions of interest (ROI’s) with respect to the original tip of the corallite is indicated. **b** Heatmaps generated from representative latitudinal cross-sectional $\mu$-CT scan images of *A. polystoma.* Differences in colour represent variation in the skeletal element thickness. HNHP = high nitrate: high phosphate, HNLP = high nitrate: low phosphate, LNHP = low nitrate: high phosphate and LNLP = low nitrate: low phosphate

**Fig. 5:**
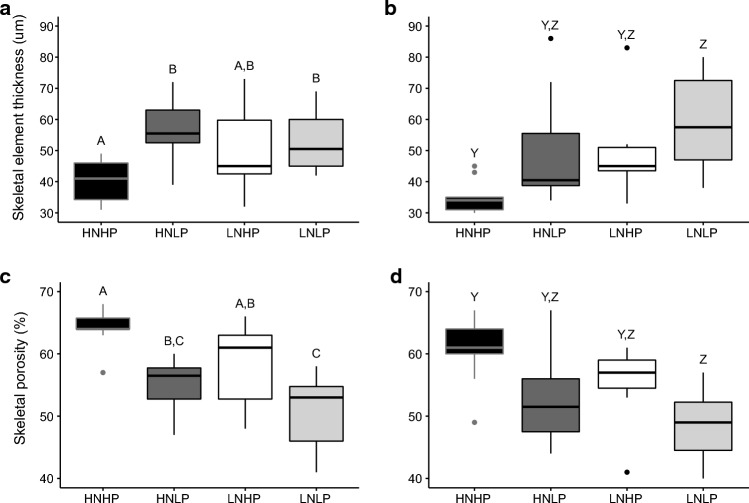
Quantitative analysis of skeletal microstructure of *A. polystoma* after culture in different nutrient environments. Mean skeletal element thickness of **a** ‘old’ skeleton (ANOVA, *p* = 0.008, *n* = 10) and **b** ‘new’ skeleton (Kruskal–Wallis, *p* = 0.006, *n* = 10). Mean skeletal porosity for **c** ‘old’ (ANOVA, p <  < 0.01, *n* = 10) and **d** ‘new’ (ANOVA, *p* = 0.006, *n* = 10) skeleton. Measurements cover ~0.25 mm thick regions of interest perpendicular to the skeletal axis. In ‘old’ skeleton, the ROI is located 0.75 to 0.50 mm below the top end of the original axial corallite, and in ‘new’ skeleton, it is located ~0.50 to 0.75 mm above the top end of the original axial corallite. Letters indicate significant differences between treatments. HNHP = high nitrate: high phosphate, HNLP = high nitrate: low phosphate and LNLP = low nitrate: low phosphate

**Fig. 6:**
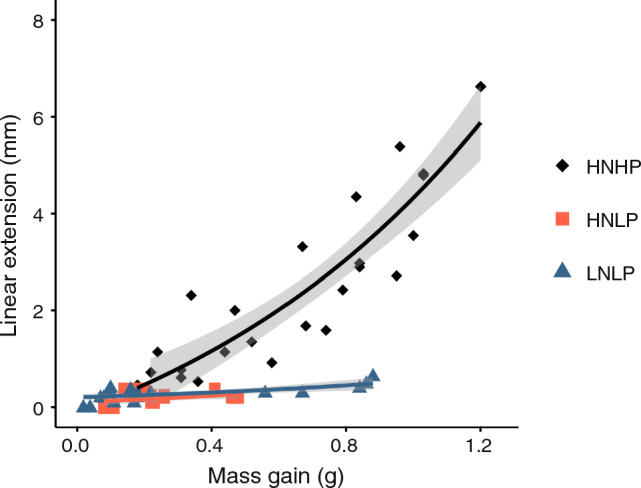
The relationship between mass gain and linear extension for *Acropora polystoma* cultured under three nutrient environments for a total of 73 d (*n* = 5). Each data point represents the linear extension and mass gain of an individual fragment at a particular time point during the culture. Data were fitted using an exponential function. The grey shading represents the 95% confidence interval for each nutrient environment. HNHP = high nitrate: high phosphate, HNLP = high nitrate: low phosphate and LNLP = low nitrate: low phosphate

The characterisation of the skeletal microstructure by X-ray micro-tomography and subsequent analysis of the resultant reconstructed 3D image files revealed a mean thickness of skeletal elements which ranged from ~30 to ~90 $\mu$m between treatments. There were statistically significant differences in mean skeletal element thickness between treatments in both ‘old’ (ANOVA, F_3,36_ = 4.627, *p* = 0.008) and ‘new’ skeleton (Kruskal–Wallis, *p* = 0.006) (Figs. 4b and 5a and b). The thinnest skeletal elements were observed in HNHP corals while significant thickening was observed in both the ‘old’ and ‘new’ skeleton of nutrient-limited (LNLP) corals. There was also significant thickening in the ‘old’ skeleton of HNLP corals while those cultured in the LNHP treatment had skeletal elements of intermediate thickness. Significant differences were also found for porosity in both ‘old’ (ANOVA, F_3,36_ = 12.4, p <  < 0.01) and ‘new’ skeleton (ANOVA, F_3,28_ = 5.175, *p* = 0.006). Porosity ranged between 41 and 67% across the treatments, being highest in HNHP corals and lowest in those from the LNLP treatment (Fig. 5c and d). Porosity was significantly reduced in the ‘old’ skeleton of HNLP corals and took intermediate values in LNHP skeletons. Across treatments, skeletal element thickness and porosity were found to be inversely related in both the ‘old’ (R^2^ = 0.39, p <  < 0.001) and ‘new’ skeleton (R^2^ = 0.46, p <  < 0.001) (supplementary material, SM Fig. 1). In corals from the HNHP treatment, mean skeletal element thickness was found to be positively correlated (R^2^ = 0.43, p <  < 0.0001) to distance from the tip of the axial corallite while in corals from the other treatments, this positive correlation was absent. Under HNHP conditions, corals showed linear extension and associated mass gain at all time points of the experiments (Fig. 6). The exponential fit of the data points suggests that relatively less mass was gained for a given unit of extension when linear extension rates were high. Contrarily, under HNLP and LNLP conditions, mass gain became largely decoupled from linear extension at later stages of the experiment.

## Discussion

Recent studies have demonstrated the sensitivity of zooxanthellate corals to skewed stoichiometries of dissolved inorganic nitrogen and phosphorus (Wiedenmann et al. 2013; D’Angelo and Wiedenmann 2014; Rosset et al. 2017). When nutrient availability remains replete with respect to both N and P, corals can sustain high symbiont densities that show high Fv/Fm values and support an increased coral tissue biomass. Some corals can withstand strong nitrogen limitation that results from phosphate enrichment with low N:P ratios, exhibiting minimal loss of symbionts, Fv/Fm and tissue mass (Rosset et al. 2017). In contrast, at high N:P ratios, the relative undersupply of phosphate leads to P-starvation of the symbionts, resulting in malfunctioning of the photosynthetic apparatus, reduced Fv/Fm and bleaching even under moderate temperature/light conditions. In their bleached appearance and reduced polyp biomass, these P-starved corals resemble those exposed to conditions in which both N and P are strongly limiting. However, in the latter case, the photosynthetic machinery of the symbionts is usually less affected and Fv/Fm values tend to stay high (Wiedenmann et al. 2013; D’Angelo and Wiedenmann 2014; Rosset et al. 2017).

The observations that corals can either respond by increasing or decreasing rates of linear extension and calcification in response to elevated concentrations of dissolved inorganic nutrients led to the notion that effects of nutrient enrichment are largely unpredictable and the involved physiological mechanisms are poorly understood (Szmant 2002). We show that it is possible to resolve some of the apparent contradictions among published studies by categorising the findings of previous studies according to the taxonomic background of the experimental corals and the N:P stoichiometry of the treatment. When the published results are grouped under these constraints, *Acropora* spp show a clear trend to respond with increased linear growth and reduced skeletal density to replete supply with N and P. In contrast, under skewed nutrient stoichiometries with high N:P ratios, growth is inhibited and skeletal density is increased. In the other genera analysed in the published studies (*Porites* spp., *Stylophora* spp., *Pocillopora* spp., *Montastrea* spp., *Montipora* spp. *Merulina* spp.), growth tends to be also inhibited by nutrient enrichment at high N:P ratios, but also combined nitrogen and phosphate enrichment caused a lower linear extension and calcification rates in a comparable number of cases. To verify the effects of skewed N:P stoichiometries on the growth and skeletal structure of *Acropora* spp., we assessed the response of *A. polystoma* to nutrient replete conditions (HNHP), strong nutrient limitation (LNLP) and the oversupply of nitrate (HNLP) and phosphate (LNHP).

Replicate fragments of *A. polystoma* exposed to HNHP conditions maintained high zooxanthellae densities with high Fv/Fm values. In contrast, corals exposed to HNLP conditions showed the distinctive symptoms of P-starvation, namely a bleached appearance associated with decreased zooxanthellae density and reduced Fv/Fm (Wiedenmann et al. 2013; Rosset et al. 2017). LNLP conditions also caused a reduction in symbiont numbers, but their Fv/Fm values were not affected, suggesting that symbiont photosynthesis remained functional despite the strong nutrient limitation (D’Angelo and Wiedenmann 2014). Corals from the LNHP treatment lost less symbionts whilst retaining high Fv/Fm values, suggesting that *A. polystoma* and its symbionts are better adapted to withstand low, rather than high N:P ratios.

The results of the present study show that nitrogen enrichment at low phosphate concentrations (HNLP treatment) and the resulting phosphate starvation (Wiedenmann et al. 2013; Rosset et al. 2017) also has profound impacts on the skeletal growth and microstructure of *A. polystoma.* Notably, nutrient enrichment at high N:P ratios has comparable effects on the skeleton as strong nutrient limitation (LNLP treatment). Specifically, linear extension and calcification are inhibited and skeletal elements thicken, leading to reduced porosity and increased density of the skeletal microstructure. The contrasted responses between the HNHP and LNLP treatments demonstrate that enrichment of both NO_3_^−^ and PO_4_^3−^ stimulates linear extension and, accordingly, calcification if both N and P are provided in sufficient amounts and in a balanced stoichiometry that does neither result in N nor P limitation or starvation. At the same time, this type of nutrient enrichment results in the formation of thinner skeletal elements and increases skeletal porosity while strong nutrient limitation has the opposite effect. An inverse correlation between extension rate and skeletal density is considered a general relationship also in several other coral species (for review see Szmant 2002). The less pronounced modification of the skeletal microstructure observed under LNHP conditions corresponds with a less severe impact of this nutrient treatment on the coral-zooxanthellae symbiosis. This observation suggests that changes to the skeletal growth and microstructure reflect the functioning of the symbiosis. The findings of the experimental study are consistent with our evaluation of the literature and the most parsimonious explanation is that *Acropora* spp. are adapted to exploit modest, and balanced N and P enrichment by increasing linear extension rates with the trade-off of a more porous skeleton. In contrast, under high N:P ratios, the zooxanthellae are affected by P-starvation, the coral becomes susceptible to bleaching and growth rates are reduced. The calcification rate seems less affected as deduced from the continued gain in coral weight (Fig. 6) so skeletal elements thicken even under these conditions. This differential response of growth and calcification to nutrient limitation can also explain the thickening of the skeletal elements under the LNHP and LNLP conditions. The greater resistance to symbiont loss under low N:P ratios likely reflects the fact that nitrogen is most commonly the limiting nutrient on coral reefs (Kleypas et al. 1999; Furnas et al. 2005, D'Angelo and Wiedenmann, 2014) and that *Acropora* spp. and their symbionts have evolved to cope with these conditions. Natural nitrate: phosphate ratios in coral reef waters are typically $\le$12:1, although this encompasses considerable spatial and temporal variability and amongst the reviewed literature. A maximum “natural” N:P ratio of ~33:1, for instance, was recorded due to nutrient enrichment through seabird guano (Savage et al. 2019). HNLP conditions with a N:P ratio of up to 74:1 have been reported for anthropogenically disturbed reefs, for instance in Brazil (Szmant 2002), Jamaica (Lapointe 1997) and Barbados and have been linked to the inhibition of skeletal growth in some non-*Acroporid* species (Spencer Davies 1990). The importance of considering all dissolved inorganic nitrogen species was demonstrated during the ENCORE experiments when the experimental addition of NH_4_^+^, resulting in N:P ratios > 70, suppressed the skeletal growth of Acroporids, while enrichment at more balanced ratios promoted linear extension (Koop et al. 2001). Critically, a recent study shows that N:P ratios of macroalgae in the Belize Barrier Reef increased from ~30:1 in the 1980s to 70:1, indicating that a skewed N:P stoichiometry coincided with dramatic reductions in live coral cover (Lapointe et al. 2021). Also, Lapointe et al. (2019) linked coral reef decline at Looe Key, Florida to an increase in N:P from 9.5 to 26.5. Our findings, alongside those of previous studies (Wiedenmann et al. 2013; Rosset et al. 2017), have identified a physiological mechanism to explain such detrimental effects on reef building corals. While the N:P stoichiometry undoubtedly plays a critical role in the nutrient physiology of symbiotic reef corals, it is important to consider also the absolute concentrations. When phosphate values in the water range around 0.3 µM, the N:P ratio seems to become less critical and the corals are likely to respond in the same way as to nutrient replete (HNHP) conditions (Rosset et al. 2017). Also, at low N concentrations < 0.7 µM in water, the impact of high N:P ratio becomes less pronounced and corals are more likely to show a strongly nutrient-limited than a phosphorus-starved phenotype (Rosset et al. 2017). Accordingly, there is a continued need for long-term data series of nutrient values in reef environments measured at sufficient frequency using suitable analytical methods with appropriate minimum detection limits (Lapointe et al. 2021).

Acroporids are important framework builders and enhance the 3-dimensional complexity of reefs, thereby supporting high levels of biodiversity and productivity. Enhanced growth rates, as observed under HNHP nutrient enrichment, may enhance the rugosity of the reef. However, since Acroporids are particularly prone to breakage (Bright et al. 2016; Puotinen et al. 2020), the high skeletal porosity associated with nutrient-fuelled fast growth will likely increase their fragmentation potential (Chamberlain 1978; Marshall 2000). While this may promote asexual propagation through resettled fragments and promote the rapid regeneration of parent colonies (Shinn 1976; Lirman 2000), frequent fragmentation may lead to a loss of genetic diversity in the population while increasing the susceptibility to specific types of predation, disease and subsequent mortality (Wallace 1985; Lirman 2000; Bright et al. 2016). Taken together, chronic nutrient enrichment, specifically with high N:P ratios, may shift the accretion/erosion balance of reefs towards net erosion, similar to the bleaching-induced inhibition of coral growth (Lange and Perry 2019; Perry et al. 2020). The consequent loss of rugosity may negatively affect ecosystem services such as fisheries, tourism income and coastal protection.

Finally, diagnostic features in the skeletal microstructure of Acroporids may be useful for interpreting the nutrient environment under which skeletons formed. Spatial variability in the density of massive coral skeletons is commonly used to identify patterns of seasonal growth and to date stress events (Fowell et al. 2016; DeCarlo and Cohen 2017) but is yet to be employed as a reliable environmental proxy in Acroporids. Positive correlations between skeletal thickness and porosity and distance from the axial corallite have previously been reported for other *Acropora* spp. (Gladfelter 1982; Roche et al. 2011). In fragments from the HNHP treatment, mean skeletal element thickness was positively correlated with distance from the axial corallite tip but this relationship was absent in fragments from the other treatments. Therefore, longitudinal density profiles may prove useful as indicators of elevated nutrient concentrations, especially if used in combination with biogeochemical markers such as skeletal $\delta$^13^C: $\delta$^18^O and/or P/Ca ratios which can be used to infer photosynthesis rates and seawater phosphate concentrations, respectively (McConnaughey 1989; LaVigne et al. 2010).

In summary, we show that the taxonomy of the corals, the N:P ratio of their dissolved inorganic nutrient environment and the P-starvation concept should be considered to resolve apparent contradictions among the published scientific literature. Underpinned by experimental evidence, our findings contribute to an improved understanding of the responses of symbiotic reef corals to changes in their nutrient environment, paving the way towards knowledge-based management of the nutrient environment in coral reefs. Specifically, our results suggest that the reef community structure and the nature of nutrient enrichment should both be considered when managing regional water quality to promote the resilience of corals to the impact of global climate change.

## Supplementary Information

Below is the link to the electronic supplementary material.

Supplementary file1 (PDF 477 kb)
