# Supplementary material for: Impact of nitrogen (N) and phosphorus (P) enrichment and skewed N:P stoichiometry on the skeletal formation and microstructure of symbiotic reef corals
Source: Coral Reefs. 2022 Apr 20;41(4):1147–59. doi: 10.1007/s00338-022-02223-0 (PMC10276130; doi:10.1007/s00338-022-02223-0)
Supplement: Supplementary file 1 — Supplementary file1 (PDF 477 kb) [file 338_2022_2223_MOESM1_ESM.pdf]

Supplementary materials

1) Supplementary Tables

Supplementary Table 1: Summary of analysed studies relating to the effects of nutrient enrichment on *Acropora spp.* The Plus symbol (+) represents a statistically significant increase in the designated metric as reported by the authors, zero (0) represents no significant effect and the minus symbol (–) represents a significant decrease. NA: Not applicable. HNHP = high nitrogen: high phosphorus, HNLP = high nitrogen: low phosphorus and LNHP = low nitrogen: high phosphorus. The list of analysed studies is given under "Supplementary References".

Reference	Species	Study type/ Location	Linear Exten-sion	Calcifi-cation	Skeletal Density	[N] by species	[P] by species	[DIN] (μM)	[DIP] (μM)	N:P ratio	Stoichio-metry class
Bongiorni et al (2003)	*Acropora eurystoma*	Field adj to fish farm/ Eilat	+	+	NA	NO_3^- = 0.385 μM<br>NO_2^- = 0.095 μM<br>NH_4^+ & NH_3 = 1.016 μM	PO_4^{3-} = 0.123 μM	1.496	0.123	12.2	HNHP
Dunn et al (2012)	*Acropora muricata*	Laboratory	+	NA	-	NO_3^- = 0.11 mg/l	PO_4^{3-} = 0.2mg/l	1.77	2.11	0.8	HNHP
Dunn et al (2012)	*Acropora muricata*	Laboratory	+	NA	-	NO_3^- = 0.13 mg/l<br>NO_2^- = 0.09 mg/l	PO_4^{3-} = 0.5 mg/l	3.54	5.26	0.7	HNHP
Savage (2019)	*Acropora formosa*	Field-undisturbed /Fiji	+	NA	NA	NO_3^- = 11.543 μM<br>NH_4^+ & NH_3 = 1.172 μM	PO_4^{3-} = 0.391 μM	12.715	0.391	32.5	HNHP
Rocker et al (2017)	*Acropora tenuis*	Field - Anthrop./ Australia	+	NA	-	DIN = 0.36 μM<br>DON = 5.53 μM	DOP = 0.15 μM<br>PO_4^{3-} = 0.09 μM	5.89	0.24	24.5	HNHP

Fabricius et al (2013)	*Acropora millepora*	Laboratory	NA	0	NA	NO_3^- = 0.26 μM<br>NH_4^+ & NH_3 = 0.22 μM<br>DON = 3.63 μM	DOP = 0.058 μM<br>PO_4^{3-} = 0.83 μM	0.48	0.83	0.58	HNHP
Fabricius et al (2013)	*Acropora millepora*	Laboratory	NA	0	NA	NO_3^- = 0.24 μM<br>NH_4^+ & NH_3 = 0.21 μM<br>DON = 7.82 μM	DOP = 0.115 μM<br>PO_4^{3-} = 1.12 μM	0.45	1.12	0.40	LNHP
Fabricius et al (2013)	*Acropora millepora*	Laboratory	NA	0	NA	NO_3^- = 0.12 μM<br>NH_4^+ & NH_3 = 0.21 μM<br>DON = 7.79 μM	DOP = 0.073 μM<br>PO_4^{3-} = 0.93 μM	0.33	0.93	0.35	LNHP
Renegar & Riegl (2005)	*Acropora cervicornis*	Laboratory	NA	0	NA	NO_3^- = 5.13 μM	PO_4^{3-} = 0.07 μM	5.13	0.07	73.3	HNLP
Renegar & Riegl (2005)	*Acropora cervicornis*	Laboratory	NA	0	NA	NO_3^- = 0.45 μM	PO_4^{3-} = 2.15 μM	0.45	2.15	0.2	LNHP
Renegar & Riegl (2005)	*Acropora cervicornis*	Laboratory	NA	-	NA	NO_3^- = 4.98 μM	PO_4^{3-} = 2.09 μM	4.98	2.09	2.4	HNHP
Renegar & Riegl (2005)	*Acropora cervicornis*	Laboratory	NA	-	NA	NO_3^- = 9.93 μM	PO_4^{3-} = 0.07 μM	9.93	0.07	142	HNLP
Renegar & Riegl (2005)	*Acropora cervicornis*	Laboratory	NA	-	NA	NO_3^- = 0. 73 μM	PO_4^{3-} = 4.14 μM	0.73	4.14	0.18	LNHP
Renegar & Riegl (2005)	*Acropora cervicornis*	Laboratory	NA	-	NA	NO_3^- = 10.01 μM	PO_4^{3-} = 4.06 μM	10.01	4.06	2.5	HNHP

Tanaka et al (2007)	*Acropora pulchra*	Laboratory	NA	+	NA	NO_3^- = 5.0 μM	PO_4^{3-} = 0.3 μM	5.0	0.3	16.7	HNHP
Koop et al (2001)	*Acropora longicyathus*	Field - experiment Australia	0	0	NA	NO_3^- = 2.94 μM NH_4^+ / NH_3 = 11.45 μM	PO_4^{3-} = 0.2 μM	14.39	0.2	72	HNLP
Koop et al (2001)	*Acropora longicyathus*	Field - experiment Australia	-	+	+	NO_3^- = 2.94 μM NH_4^+ / NH_3 = 36.2 μM	PO_4^{3-} = 0.16 μM	39.14	0.16	245	HNLP
Koop et al (2001)	*Acropora longicyathus*	Field - experiment Australia	0	0	NA	NO_3^- = 2.94 μM NH_4^+ / NH_3 = 0.65 μM	PO_4^{3-} = 2.34 μM	3.59	2.34	1.5	HNHP
Koop et al (2001)	*Acropora longicyathus*	Field - experiment Australia	+	+	-	NO_3^- = 2.94 μM NH_4^+ / NH_3 = 0.65 μM	PO_4^{3-} = 5.14 μM	3.59	5.14	0.7	HNHP
Koop et al (2001)	*Acropora longicyathus*	Field - experiment Australia	0	0	NA	NO_3^- = 2.94 μM NH_4^+ / NH_3 = 11.45 μM	PO_4^{3-} = 2.34 μM	14.39	2.34	6.1	HNHP
Koop et al (2001)	*Acropora longicyathus*	Field - experiment Australia	+	+	NA	NO_3^- = 2.94 μM NH_4^+ / NH_3 = 36.2 μM	PO_4^{3-} = 5.14 μM	39.14	5.14	7.6	HNHP
Koop et al (2001)	*Acropora palifera*	Field - experiment Australia	0	NA	NA	NO_3^- = 2.94 μM NH_4^+ / NH_3 = 11.45 μM	PO_4^{3-} = 0.2 μM	14.39	0.2	72	HNLP
Koop et al (2001)	*Acropora palifera*	Field - experiment Australia	-	-	NA	NO_3^- = 2.94 μM NH_4^+ / NH_3 = 36.2 μM	PO_4^{3-} = 0.16 μM	39.14	0.16	245	HNLP
Koop et al (2001)	*Acropora palifera*	Field - experiment Australia	0	NA	NA	NO_3^- = 2.94 μM NH_4^+ / NH_3 = 0.65 μM	PO_4^{3-} = 2.34 μM	3.59	2.34	1.5	HNHP

Koop et al (2001)	*Acropora palifera*	Field - experiment Australia	+	+	NA	NO_3^- = 2.94 µM NH_4^+ / NH_3 = 0.65 µM	PO_4^{3-} = 5.14 µM	3.59	5.14	0.7	HNHP
Koop et al (2001)	*Acropora palifera*	Field - experiment Australia	0	NA	NA	NO_3^- = 2.94 µM NH_4^+ / NH_3 = 11.45 µM	PO_4^{3-} = 2.34 µM	14.39	2.34	6.1	HNHP
Koop et al (2001)	*Acropora palifera*	Field - experiment Australia	+	0	NA	NO_3^- = 2.94 µM NH_4^+ / NH_3 = 36.2 µM	PO_4^{3-} = 5.14 µM	39.14	5.14	7.6	HNHP
Koop et al (2001)	*Acropora aspera*	Field - experiment Australia	NA	NA	NA	NO_3^- = 2.94 µM NH_4^+ / NH_3 = 11.45 µM	PO_4^{3-} = 0.2 µM	14.39	0.2	72	HNLP
Koop et al (2001)	*Acropora aspera*	Field - experiment Australia	NA	0	NA	NO_3^- = 2.94 µM NH_4^+ / NH_3 = 36.2 µM	PO_4^{3-} = 0.16 µM	39.14	0.16	245	HNLP
Koop et al (2001)	*Acropora aspera*	Field - experiment Australia	NA	NA	NA	NO_3^- = 2.94 µM NH_4^+ / NH_3 = 0.65 µM	PO_4^{3-} = 2.34 µM	3.59	2.34	1.5	HNHP
Koop et al (2001)	*Acropora aspera*	Field - experiment Australia	NA	-	NA	NO_3^- = 2.94 µM NH_4^+ / NH_3 = 0.65 µM	PO_4^{3-} = 5.14 µM	3.59	5.14	0.7	HNHP
Koop et al (2001)	*Acropora aspera*	Field - experiment Australia	NA	NA	NA	NO_3^- = 2.94 µM NH_4^+ / NH_3 = 11.45 µM	PO_4^{3-} = 2.34 µM	14.39	2.34	6.1	HNHP
Koop et al (2001)	*Acropora aspera*	Field - experiment Australia	NA	0	NA	NO_3^- = 2.94 µM NH_4^+ / NH_3 = 36.2 µM	PO_4^{3-} = 5.14 µM	39.14	5.14	7.6	HNHP

Supplementary Table 2: Summary of reviewed studies relating to the effects of nutrient enrichment of species other than *Acropora* spp. Plus symbol (+) represents a statistically significant increase in the designated metric as reported by the authors, zero (0) represents no significant effect and the minus symbol (–) represents a significant decrease. NA: Not applicable. HNHP = high nitrogen: high phosphorus, HNLP = high nitrogen: low phosphorus and LNHP = low nitrogen: high phosphorus. The list of analysed studies is given under "Supplementary References".

**Reference**	**Species**	**Study type/ Location**	**Linear Extension**	**Calcification**	**Skeletal Density**	**[N] by species**	**[P] by species**	**DIN (μM)**	**DIP (μM)**	**N:P ratio**	**Stoichiometry class**
Bongiorni et al (2003)	*Stylophora pistillata*	Field close to fish farm/ Eilat	+	NA	NA	NO_3^- = 0.385 μM NO_2^- = 0.095 μM NH_4^+ & NH_3 = 1.016 μM	PO_4^{3-} = 0.123 μM	1.496	0.123	12.2	HNHP
Dikou (2009)	*Merulina ampliata*	Field – Natural/ Nutrient /sediment gradient	-	NA	NA	NO_3^- = 40 ppb	PO_4^{3-} = 15 ppb	0.65	0.16	4.1	HNHP
Edinger et al (2000)	*Porites lobata*	Field - Anthrop./ Indonesia	-	NA	NA	NO_3^- = 1.46 μM	PO_4^{3-} = 0.46 μM	1.46	0.46	3.2	HNHP
Elizalde-Rendon et al (2010)	*Porites astreoides*	Field - Anthrop./ Caribbean	-	-	-	NO_3^- = 14.65 μM	PO_4^{3-} = 2.8 μM	14.65	2.8	5.2	HNHP
Jompa & McCook (2002)	*Porites cylindrica*	Field - experiment Australia	0	NA	NA	NH_4^+ & NH_3 = 10 μM	PO_4^{3-} = 1.0 μM	10	1	10	HNHP
Marubini et al (1999)	*Porites porites*	Laboratory	-	-	NA	NO_3^- = 20.2 μM	PO_4^{3-} = 0.05 μM	20.2	0.05	404	HNLP

Marubini et al (1999)	*Porites porites*	Laboratory	-	-	NA	NH_4^+ & NH_3 = 20.2 µM	PO_4^{3-} = 0.05 µM	20.2	0.05	404	HNLP
Meyer & Schultz (1985)	*Poites furcata*	Field adj. to fish farm/ US Virgin I.	0	+	0	DON = 3.9 mmol m^2	PO_4^{3-} = 0.2 mmol m^2	3.9	0.2	19.5	HNHP
Stambler et al (1991)	*Pocillipora damicornis*	Laboratory	0	NA	NA	NH_4^+ & NH_3 = 2.0 µM	PO_4^{3-} = 2.0 µM	2.0	2.0	1	HNHP
Stambler et al (1991)	*Pocillipora damicornis*	Laboratory	0	NA	NA	NH_4^+ & NH_3 = 2.0 µM	PO_4^{3-} = 0.5 µM	2.0	0.5	4	HNHP
Stambler et al (1991)	*Pocillipora damicornis*	Laboratory	0	NA	NA	NH_4^+ & NH_3 = 7.0 µM	PO_4^{3-} = 0.1 µM	7.0	0.1	70	HNLP
Stambler et al (1991)	*Pocillipora damicornis*	Laboratory	-	NA	NA	NH_4^+ & NH_3 = 15 µM	PO_4^{3-} = 0.1 µM	15	0.1	150	HNLP
Stambler et al (1991)	*Pocillipora damicornis*	Laboratory	-	NA	NA	NH_4^+ & NH_3 = 15 µM	PO_4^{3-} = 0.5 µM	15	0.5	30	HNHP
Stambler et al (1991)	*Pocillipora damicornis*	Laboratory	-	NA	NA	NH_4^+ & NH_3 = 15 µM	PO_4^{3-} = 2.0 µM	15	2.0	7.5	HNHP
Tomascik & Sander (1985)	*Montastrea annularis*	Field – Anthrop./ Barbados	-	NA	NA	NO_2 & NO_3^- - N = 0.816 ug l<br>NH_4^+ & NH_3 – N = 0.976 ug l	PO_4^{3-} - P = 0.103 ug l	0.128	0.0033	38.8	HNLP

Tomascik & Sander (1985)	*Montastrea annularis*	Field – Anthrop./ Barbados	-	NA	NA	NO_2 & NO_3^- - N = 4.424 ug l<br>NH_4^+ & NH_3 – N = 2.695 ug l	PO_4^{3-} - P = 0.214 ug l	0.5085	0.0069	73.7	HNLP
Fabricius et al (2013)	*Montipora tuberculosa*	Laboratory	NA	0	NA	NO_3^- & NO_2^- = 0.26 μM<br>NH_4^+ = 0.22 μM<br>PON = 3.63 μM	SRP = 0.058 μM<br>POP = 0.83 μM	0.48	0.83	0.58	HNHP
Fabricius et al (2013)	*Montipora tuberculosa*	Laboratory	NA	-	NA	NO_3^- & NO_2^- = 0.24 μM<br>NH_4^+ = 0.21 μM<br>PON = 7.79 μM	SRP = 0.115 μM<br>POP = 1.12 μM	0.45	1.12	0.40	LNHP
Fabricius et al (2013)	*Montipora tuberculosa*	Laboratory	NA	0	NA	NO_3^- & NO_2^- = 0.12 μM<br>NH_4^+ = 0.21 μM<br>PON = 7.82 μM	SRP = 0.073 μM<br>POP = 0.93 μM	0.33	0.93	0.35	LNHP
Ferrier-Pages et al (2000)	*Stylophora pistillata*	Laboratory	NA	-	NA	NH_4^+ & NH_3 = 9 μM	PO_4^{3-} = 0.2 μM	9	0.2	45	HNLP
Ferrier-Pages et al (2000)	*Stylophora pistillata*	Laboratory	NA	-	NA	NH_4^+ & NH_3 = 0.4 μM	PO_4^{3-} = 2.8 μM	0.4	2.8	0.1	LNHP
Ferrier-Pages et al (2000)	*Stylophora pistillata*	Laboratory	NA	-	NA	NH_4^+ & NH_3 = 8.5 μM	PO_4^{3-} = 2.4 μM	8.5	2.4	3.5	HNHP
Ferrier-Pages et al (2000)	*Stylophora pistillata*	Laboratory	NA	-	NA	NH_4^+ & NH_3 = 19.5 μM	PO_4^{3-} = 0.2 μM	19.5	0.2	97.5	HNLP

Ferrier-Pages et al (2000)	*Stylophora pistillata*	Laboratory	NA	-	NA	NH_4^+ & NH_3 = 0.4 μM	PO_4^{3-} = 2.8 μM	0.4	2.8	0.1	LNHP
Ferrier-Pages et al (2000)	*Stylophora pistillata*	Laboratory	NA	-	NA	NH_4^+ & NH_3 = 18.5 μM	PO_4^{3-} = 2.4 μM	18.5	2.4	7.7	HNHP
Ferrier-Pages et al (2001)	*Stylophora pistillata*	Laboratory	NA	-	NA	NO_3^- = 2.5 μM<br>NH_4^+ & NH_3 = 0.4 μM	PO_4^{3-} = 0.2 μM	2.9	0.2	14.5	HNHP
Godinot et al (2011)	*Stylophora pistillata*	Laboratory	NA	0	NA	NO_3^- = 0.4 μM<br>NH_4^+ & NH_3 = 0.5 μM	PO_4^{3-} = 0.5 μM	0.9	0.5	1.8	HNHP
Godinot et al (2011)	*Stylophora pistillata*	Laboratory	NA	+	NA	NO_3^- = 0.4 μM<br>NH_4^+ & NH_3 = 0.5 μM	PO_4^{3-} = 0.5 μM	0.9	2.5	0.4	LNHP
Marubini & Davies (1996)	*Porites porites*	Laboratory	NA	-	NA	NO_3^- = 1.0 μM	PO_4^{3-} = 2.5 μM	1.0	0.05	20	HNHP
Marubini & Davies (1996)	*Porites porites*	Laboratory	NA	-	NA	NO_3^- = 5.0 μM	PO_4^{3-} = 0.5 μM	5.0	0.05	100	HNLP
Marubini & Davies (1996)	*Porites porites*	Laboratory	NA	-	NA	NO_3^- = 20 μM	PO_4^{3-} = 0.5 μM	20	0.05	400	HNLP
Marubini & Davies (1996)	*Montastrea annularis*	Laboratory	NA	-	NA	NO_3^- = 1.0 μM	PO_4^{3-} = 2.5 μM	1.0	0.05	20	HNHP
Marubini & Davies (1996)	*Montastrea annularis*	Laboratory	NA	-	NA	NO_3^- = 5.0 μM	PO_4^{3-} = 0.5 μM	5.0	0.05	100	HNLP

Marubini & Davies (1996)	*Montastrea annularis*	Laboratory	NA	-	NA	NO_3^- = 20 µM	PO_4^{3-} = 0.5 µM	20	0.05	400	HNLP
Marubini & Atkinson (1999)	*Porites compressa*	Laboratory	NA	0	NA	NO_3^- = 0.91 µM NH_4^+ & NH_3 = 0.41 µM	PO_4^{3-} = 0.13 µM	1.32	0.13	10.2	HNHP
Marubini & Atkinson (1999)	*Porites compressa*	Laboratory	NA	0	NA	NO_3^- = 1.48 µM NH_4^+ & NH_3 = 0.46 µM	PO_4^{3-} = 0.12 µM	1.94	0.12	16.2	HNHP
Marubini & Atkinson (1999)	*Porites compressa*	Laboratory	NA	0	NA	NO_3^- = 5.66 µM NH_4^+ & NH_3 = 0.42 µM	PO_4^{3-} = 0.11 µM	6.08	0.11	55	HNLP
Meyer et al (1983)	*Porites furcata*	Field-natural/US Virgin I.	NA	+	NA	DON = 3.9 mmol m^2	PO_4^{3-} = 0.2 mmol m^2	3.9	0.2	19.5	HNHP
Silbiger et al (2018)	*Porites compressa, Montipora capitata*	Laboratory	NA	-	NA	NO_3^- = 3.6 µM	PO_4^{3-} = 1.08 µM	3.6	1.08	3.3	HNHP
Silbiger et al (2018)	*Porites compressa, Montipora capitata*	Laboratory	NA	-	NA	NO_3^- = 7.61 µM	PO_4^{3-} = 2.6 µM	7.61	2.6	2.9	HNHP
Spencer Davies (1990)	Porites porites	Field-Anthrop./ Barbados	NA	0	NA	NO_3^- = 4.92 ug l	PO_4^{3-} = 0.17 ug l	0.0794	0.00179	44	HNLP
Spencer Davies (1990)	Montastrea annularis	Field-Anthrop./ Barbados	NA	-	NA	NO_3^- = 4.92 ug l	PO_4^{3-} = 0.17 ug l	0.0794	0.00179	44	HNLP

Tanaka et al (2017)	*Montipora digitata*	Laboratory	NA	0	NA	NO_3^- = 1.86 μM NO_2^- = 0.14 μM NH_4^+ & NH_3 = 0.02 μM	PO_4^{3-} = 0.02 μM	2.02	0.02	101	HNLP
Tanaka et al (2017)	*Montipora digitata*	Laboratory	NA	0	NA	NO_3^- = 1.37 μM NO_2^- = 0.11 μM NH_4^+ & NH_3 = 0.02 μM	PO_4^{3-} = 0.12 μM	1.50	0.12	12.5	HNHP
Tanaka et al (2017)	*Porites cylindrica*	Laboratory	NA	0	NA	NO_3^- = 1.73 μM NO_2^- = 0.14 μM NH_4^+ & NH_3 = 0.03 μM	PO_4^{3-} = 0.03 μM	1.90	0.03	63	HNLP
Tanaka et al (2017)	*Porites cylindrica*	Laboratory	NA	0	NA	NO_3^- = 1.46 μM NO_2^- = 0.12 μM NH_4^+ & NH_3 = 0.04 μM	PO_4^{3-} = 0.12 μM	1.62	0.12	13.5	HNHP
Koop et al (2001)	*Stylophora pistillata*	Field - experiment Australia	NA	NA	NA	NO_3^- = 2.94 μM NH_4^+ / NH_3 = 11.45 μM	PO_4^{3-} = 0.2 μM	14.39	0.2	72	HNLP
Koop et al (2001)	*Stylophora pistillata*	Field - experiment Australia	NA	-	NA	NO_3^- = 2.94 μM NH_4^+ / NH_3 = 36.2 μM	PO_4^{3-} = 0.16 μM	39.14	0.16	245	HNLP
Koop et al (2001)	*Stylophora pistillata*	Field - experiment Australia	NA	NA	NA	NO_3^- = 2.94 μM NH_4^+ / NH_3 = 0.65 μM	PO_4^{3-} = 2.34 μM	3.59	2.34	1.5	HNHP
Koop et al (2001)	*Stylophora pistillata*	Field - experiment Australia	NA	0	NA	NO_3^- = 2.94 μM NH_4^+ / NH_3 = 0.65 μM	PO_4^{3-} = 5.14 μM	3.59	5.14	0.7	HNHP
Koop et al (2001)	*Stylophora pistillata*	Field - experiment Australia	NA	NA	NA	NO_3^- = 2.94 μM NH_4^+ / NH_3 = 11.45 μM	PO_4^{3-} = 2.34 μM	14.39	2.34	6.1	HNHP

Koop et al (2001)	*Stylophora pistillata*	Field - experiment Australia	NA	0	NA	NO_3^- = 2.94 μM NH_4^+ / NH_3 = 36.2 μM	PO_4^{3-} = 5.14 μM	39.14	5.14	7.6	HNHP
Koop et al (2001)	*Pocillipora damicornis*	Field - experiment Australia	NA	0	NA	NO_3^- = 2.94 μM NH_4^+ / NH_3 = 11.45 μM	PO_4^{3-} = 0.2 μM	14.39	0.2	72	HNLP
Koop et al (2001)	*Pocillipora damicornis*	Field - experiment Australia	NA	-	NA	NO_3^- = 2.94 μM NH_4^+ / NH_3 = 36.2 μM	PO_4^{3-} = 0.16 μM	39.14	0.16	245	HNLP
Koop et al (2001)	*Pocillipora damicornis*	Field - experiment Australia	NA	0	NA	NO_3^- = 2.94 μM NH_4^+ / NH_3 = 0.65 μM	PO_4^{3-} = 2.34 μM	3.59	2.34	1.5	HNHP
Koop et al (2001)	*Pocillipora damicornis*	Field - experiment Australia	NA	0	NA	NO_3^- = 2.94 μM NH_4^+ / NH_3 = 0.65 μM	PO_4^{3-} = 5.14 μM	3.59	5.14	0.7	HNHP
Koop et al (2001)	*Pocillipora damicornis*	Field - experiment Australia	NA	0	NA	NO_3^- = 2.94 μM NH_4^+ / NH_3 = 11.45 μM	PO_4^{3-} = 2.34 μM	14.39	2.34	6.1	HNHP
Koop et al (2001)	*Pocillipora damicornis*	Field - experiment Australia	NA	-	NA	NO_3^- = 2.94 μM NH_4^+ / NH_3 = 36.2 μM	PO_4^{3-} = 5.14 μM	39.14	5.14	7.6	HNHP

2) Supplementary Figure

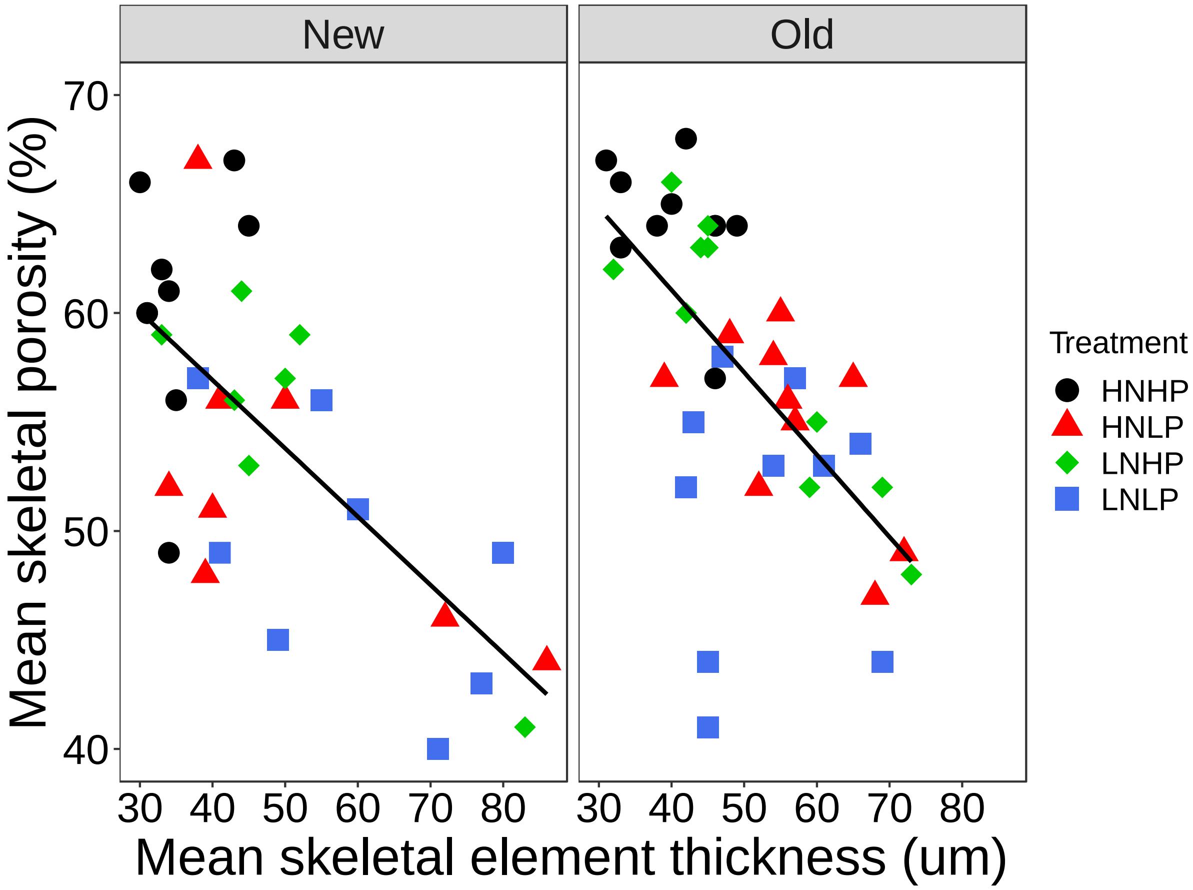

Supplementary Figure 1: The relationship between mean skeletal element thickness and mean skeletal porosity in *Acropora polystoma* for 'new' and 'old' skeleton when cultured under 4 different nutrient treatments for > 10 weeks. Each point represents a measurement within the region of interest (see main text) for a single coral fragment. Statistically significant correlations were found in both the 'new' ($R^2 = 0.46$, $p << 0.001$) and 'old' skeleton ($R^2 = 0.39$, $p << 0.001$). HNHP = high nitrate: high phosphate, HNLP = high nitrate: low phosphate, LNHP = low nitrate: high phosphate and LNLP = low nitrate: low phosphate.

3) Supplementary Methods

Macro used in ImageJ for analysis of CT scan slices

The following Macro was utilized for all measurements relating to “skeletal element thickness” and “porosity”. Prior to running the macro an individual slice within the region of interest was selected. The macro relies upon the BoneJ plug-in being installed.

```
run("3D Project...", "projection=[Brightest Point] axis=Y-Axis slice=1 initial=0 total=360 rotation=10 lower=1 upper=255 opacity=0 surface=100 interior=50");
setOption("BlackBackground", false);
run("Make Binary", "method=Intermodes background=Dark calculate");
run("Thickness", "thickness mask");
run("Volume Fraction", "algorithm=Voxel surface=6 use");
setOption("BlackBackground", false);
run("Dilate", "stack");
run("Dilate", "stack");
run("Dilate", "stack");
run("Dilate", "stack");
run("Dilate", "stack");
run("Fill Holes", "stack");
run("Erode", "stack");
run("Erode", "stack");
run("Erode", "stack");
run("Erode", "stack");
run("Erode", "stack");
run("Volume Fraction", "algorithm=Voxel surface=6 use");
String.copyResults();
String.copyResults();
```

4) Supplementary References

Bongiorni L, Shafir S, Angel D, Rinkevich B (2001) Survival, growth and gonad development of two hermatypic corals subjected to in situ fish-farm nutrient enrichment. Mar Ecol Prog Ser. 253:137–44.

Browne NK, Tay JKL, Low J, Larson O, Todd PA (2015) Fluctuations in coral health of four common inshore reef corals in response to seasonal and anthropogenic changes in water quality. Mar Environ Res 105:39–52.

Dikou A (2009) Skeletal linear extension rates of the foliose scleractinian coral Merulina ampliata (Ellis & Solander, 1786) in a turbid environment. Mar Ecol. 30:405–15.

Dunn JG, Sammarco PW, LaFleur G (2012) Effects of phosphate on growth and skeletal density in the scleractinian coral Acropora muricata: A controlled experimental approach. J Exp Mar Bio Ecol. 411:34–44.

Edinger EN, Limmon G V., Jompa J, Widjatmoko W, Heikoop JM, Risk MJ (2000) Normal Coral Growth Rates on Dying Reefs: Are Coral Growth Rates Good Indicators of Reef Health? Mar Pollut Bull. 40:404–25.

Elizalde-Rendón EM, Horta-Puga G, González-Diaz P, Carricart-Ganivet JP (2010) Growth characteristics of the reef-building coral Porites astreoides under different environmental conditions in the Western Atlantic. Coral Reefs. 29:607–14.

Fabricius KE, Cséke S, Humphrey C, De'ath G (2013) Does Trophic Status Enhance or Reduce the Thermal Tolerance of Scleractinian Corals? A Review, Experiment and Conceptual Framework. PLoS One. 8:1–12.

Ferrier-Pagès C, Schoelzke V, Jaubert J, Muscatine L, Hoegh-Guldberg O (2001) Response of a scleractinian coral, Stylophora pistillata, to iron and nitrate enrichment. J Exp Mar Bio Ecol. 259249–61.

Ferrier-Pagès C, Gattuso JP, Dallot S, Jaubert J (2000) Effect of nutrient enrichment on growth and photosynthesis of the zooxanthellate coral Stylophora pistillata. Coral Reefs. 19:103–13.

Godinot C, Ferrier-Pagès C, Montagna P, Grover R (2011) Tissue and skeletal changes in the scleractinian coral Stylophora pistillata Esper 1797 under phosphate enrichment. J Exp Mar Bio Ecol 409:200–7.

Jompa J, McCook LJ (2002) The effects of nutrients and herbivory on competition between a hard coral (Porites cylindrica) and a brown alga (Lobophora variegata). Limnol Oceanogr. 47:527–34.

Koop K, Booth D, Broadbent A, Brodie J, Bucher D, Capone D, Coll J, Dennison W, Erdmann M, Harrison P, Hoegh-Guldberg O, Hutchings P, Jones GB, Larkum AWD, O'Neil J, Steven A, Tentori E, Ward S, Williamson J, Yellowlees D (2001) ENCORE: The effect of nutrient enrichment on coral reefs. Synthesis of results and conclusions. Mar Pollut Bull 42:91–120

Marubini F, Atkinson MJ (1999) Effects of lowered pH and elevated nitrate on coral calcification. Mar Ecol Prog Ser. 188:117–21.

Marubini F, Davies PS (1996) Nitrate increases zooxanthellae population density and reduces skeletogenesis in corals. Mar Biol. 127:319–28.

Marubini F, Thake B, May P (1999) Bicarbonate Addition Promotes Coral Growth. Limnol Oceanogr. 44:716–20.

Meyer JL, Schultz ET (1985) Tissue Condition and Growth Rate of Corals Associated with Schooling Fish. Limnol Oceanogr. 30:157–66.

Meyer JL, Schultz ET, Helfman GS (1983) Fish Schools : An Asset to Corals. Science 220:1047–9.

Renegar DA, Riegl BM (2005) Effect of nutrient enrichment and elevated CO2 partial pressure on growth rate of Atlantic scleractinian coral Acropora cervicornis. Mar Ecol Prog Ser. 293:69–76.

Riegl B, Johnston M, Glynn PW, Keith I, Rivera F, Vera-Zambrano M, Banks, S, Feingold, J, Glynn, PJ (2019) Some environmental and biological determinants of coral richness, resilience and reef building in Galápagos (Ecuador). Sci Rep 9:1–16.

Rocker MM, Francis DS, Fabricius KE, Willis BL, Bay LK (2017) Variation in the health and biochemical condition of the coral Acropora tenuis along two water quality gradients on the Great Barrier Reef , Australia. Mar Pollut Bull 119:106–19.

Savage C (2019) Seabird nutrients are assimilated by corals and enhance coral growth rates. Sci Rep 9:4284.

Silbiger NJ, Nelson CE, Remple K, Sevilla JK, Quinlan ZA, Putnam HM, Fox MD, Donahue MJ (2018) Nutrient pollution disrupts key ecosystem functions on coral reefs. Proc R Soc B Biol Sci. 285:2–10.

Spencer Davies P (1990) A rapid method for assessing growth rates of corals in relation to water pollution. Mar Pollut Bull. 21:346–8.

Stambler N, Popper N, Dubinsky Z, Stimson J (1991) Effects of nutrient enrichment and water motion on the coral Pocillopora damicornis. Pacific Sci. 45:299–307.

Tanaka Y, Grottoli AG, Matsui Y, Suzuki A, Sakai K (2017) Effects of nitrate and phosphate availability on the tissues and carbonate skeleton of scleractinian corals. Mar Ecol Prog Ser. 570:101–12.

Tanaka Y, Miyajima T, Koike I, Hayashibara T, Ogawa H (2007) Imbalanced coral growth between organic tissue and carbonate skeleton caused by nutrient enrichment. Limnol Oceanogr. 521139–46.

Tomascik T (1990) Growth rates of two morphotypes of Montastrea annularis along a eutrophication gradient, Barbados, W.I. Mar Pollut Bull. 21:376–81.

Tomascik T, Sander F (1985) Effects of eutrophication on reef-building corals. Mar Biol. 87:143–55.
